# Association between the quality of plant‐based diets and risk of frailty

**DOI:** 10.1002/jcsm.13077

**Published:** 2022-09-30

**Authors:** Mercedes Sotos‐Prieto, Ellen A. Struijk, Teresa T. Fung, Fernando Rodríguez‐Artalejo, Walter C. Willett, Frank B. Hu, Esther Lopez‐Garcia

**Affiliations:** ^1^ Department of Preventive Medicine and Public Health, School of Medicine Autonomous University of Madrid Madrid Spain; ^2^ CIBERESP (CIBER of Epidemiology and Public Health) Madrid Spain; ^3^ Department of Environmental Health Harvard T.H. Chan School of Public Health Boston MA USA; ^4^ IMDEA‐Food Institute CEI UAM+CSIC Madrid Spain; ^5^ Department of Nutrition Simmons University Boston MA USA; ^6^ Department of Nutrition Harvard T.H. Chan School of Public Health Boston MA USA; ^7^ Channing Division of Network Medicine, Department of Medicine Brigham and Women's Hospital, Harvard Medical School Boston MA USA

**Keywords:** plant‐based diet, diet quality, frailty, older adults, cohort study

## Abstract

**Background:**

The Mediterranean diet and other dietary patterns rich in fruits and vegetables have been linked to lower risk of frailty in older adults. However, not all plant‐based diets are necessarily healthful, and no previous study has evaluated the role of the quality of plant‐based dietary patterns in frailty risk. Our aim was to assess the association between plant‐based diet quality and risk of frailty.

**Methods:**

Prospective cohort consisted with 82 234 women aged ≥60 years from the Nurses' Health Study, who were followed from 1990 through 2014. The dates of analysis were April 14 to June 23, 2021. Dietary data were collected every 4 years using a validated semi‐quantitative food frequency questionnaire. The plant‐based diet quality was assessed with two indices (range 18–90 points): (a) healthful plant‐based diet index (hPDI), where healthy plant foods (whole grains, fruits, vegetables, nuts, legumes, vegetable oils and tea/coffee) received positive scores, while less healthy plant foods (fruit juices, sweetened beverages, refined grains, potatoes, and sweets/desserts) and animal foods received reverse scores; and (b) unhealthful plant‐based diet index (uPDI) where positive scores were given to less healthy plant foods and reverse scores to healthy plant foods and animal foods. Frailty incidence was assessed every 4 years, being defined as having three or more of the following five criteria from the FRAIL scale: fatigue, low strength, reduced aerobic capacity, having ≥5 illnesses and weight loss ≥5%. Multivariable‐adjusted Cox proportional‐hazards models were used to estimate hazard ratios (HRs) and their 95% confidence interval (CI).

**Results:**

We identified 12 910 incident cases of frailty over 1 176 401 person‐year follow‐up. In the multivariable analysis, the hPDI was inversely associated with the risk of frailty (hazard ratio [HR] for the highest vs. lowest quintile: 0.77, 95% confidence interval: 0.72–0.81; *P* trend <0.001). In addition, a 10‐unit increment in the hPDI was associated with a relative 15% lower risk of frailty. Conversely, a direct association was found between the uPDI and risk of frailty (HR highest vs. lowest quintile: 1.24 [1.17, 1.32], *P* trend <0.001). These associations were consistent for each frailty criterion, among participants with no frailty criteria at baseline, after excluding participants with diabetes, cancer and cardiovascular disease at baseline, for alternative versions of the plant‐based diet indices (PDIs), in subgroup analysis by categories of potential confounders, and in latency analysis.

**Conclusions:**

A healthful plant‐based diet was associated with lower risk of frailty whereas an unhealthful plant‐based diet was associated with higher risk.

## Introduction

Plant‐based diets are characterized by high consumption of plant foods and low or no intake of animal food. They have recently gained attention due to their increasingly recognized health effects.^1^ In addition, the EAT‐Lancet report recommends a global shift towards plant‐based diets for environmental sustainability^2^ and the Dietary Guidelines of Americans 2020–2025 included a recommendation for consuming a Healthy Vegetarian Dietary Pattern to prevent chronic diseases.^3^ However, vegetarian diets have raised concern about possible nutrient deficiencies associated with their habitual consumption, affecting vitamin B12 and other vitamins. This is especially important in older adults, because nutrition deficiencies have been associated in a bidirectional way with higher risk of frailty.^4^, ^5^ Sources of proteins (animal vs protein) and risk of frailty have also been studied with inconsistent results. However, a recent study found that women with a higher intake of plant protein had a lower risk of frailty.^6^

Frailty is a frequent geriatric syndrome characterized by reduced physiological reserve^7^ that entails a higher risk of adverse health‐related outcomes, including hospitalization, falls, disability and death.^8^ Previous studies have reported an association of several high‐quality diets rich in fruits and vegetables, such as the Mediterranean diet, the DASH diet or a diet represented by the alternate healthy eating index, with lower risk of frailty.^9^, ^10^, ^11^, ^12^

However, not all diets rich in plant‐based foods are necessarily healthful. Indeed, the quality of their food components is notoriously important, because refined carbohydrates, added sugars or ultra‐processed foods are negatively associated with chronic diseases,^1^, ^13^ whereas whole grains, fruits, vegetables and plant‐based proteins from legumes, nuts and tofu show a beneficial effect.^1^, ^14^ With the aim to reflect the quality of plant‐based diets, two separate plant‐based diet indices has been developed: a healthful plant‐ based diet (hPDI), characterized by healthy plant foods (whole grains, fruits, vegetables, nuts, legumes, vegetable oils and tea and coffee); and an unhealthful PDI (uPDI) characterized by less healthy plant foods (fruit juices, refined grains, potatoes, sugar‐sweetened beverages, sweets and desserts).^15^, ^16^ This approach, unlike others that consider only yes/no items to describe vegetarians versus non‐vegetarians dietary patterns, is able to better capture the quality of the plant‐based diets. In previous studies, these two PDIs have been associated with the risk of cardiovascular disease, diabetes, other chronic diseases and mortality.^17^, ^18^, ^19^, ^20^

In the Nurses' Health Study, we previously found that higher consumption of fruits and vegetables was related to lower risk of frailty,^21^ and by contrast, higher consumption of sugar‐sweetened beverages was associated with higher frailty risk.^22^ However, no previous research has evaluated the role of the quality of plant‐based dietary patterns in frailty risk. Therefore, our aim is to assess the association between plant‐based diet quality and risk of frailty among women in the Nurses' Health Study.

## Methods

### Study population

The Nurses' Health Study (NHS) is an ongoing study of 121 700 female nurses aged 30–55 years in 1976, when this study began.^23^ Every 2 years, participants provided information about medical history and health‐related factors. Follow‐up rates exceed 90%. For this analysis, the baseline was set at 1992, when frailty was first calculated, and the follow‐up went through 2014. Also, for this analysis, we included women aged ≥60 years in 1992 and those who turned 60 years thereafter, with valid dietary information (>500 kcal/day and <3500 kcal/day) during the follow‐up. We excluded women who had prevalent frailty at baseline, leaving 82 234 women for the analysis (*Figure* *S1*).

The NHS protocol was approved by the institutional review boards of the Brigham and Women's Hospital and Harvard T.H. Chan School of Public Health, and study participants provided written informed consent.

### Assessment of plant‐based diet indices

Dietary intake was assessed every 4 years using a validated food frequency questionnaire (FFQ)^24^ (1990, 1994, 1998, 2002, 2006 and 2010). Participants were asked how often on average during the previous year they had consumed each food of a standard portion size. Reliability and validity of the FFQ have been reported elsewhere.^25^

The two types of plant‐based diet indices, the hPDI and uPDI, has been described elsewhere.^15^, ^16^ Briefly, 18 food groups based on nutrients and culinary similarities were created within the larger categories of (1) healthy plant foods (whole grains, fruits, vegetables, nuts, legumes, vegetable oils and tea/coffee); (2) less healthy plant foods (fruit juices, refined grains, potatoes, sugar‐sweetened beverages and sweets/desserts); and (3) animal foods (animal fat, dairy, eggs, fish/seafood, meat and miscellaneous animal‐based foods). The 18 food groups were ranked into quintiles, and each quintile was given a positive or reverse score, with a range from 1 to 5 (*Table* *S1*). For the hPDI, the healthy plant food groups were given positive scores whereas the less healthy plant food and animal food groups were given a reverse score. As regards the uPDI, positive scores were given to less healthy plant food groups, and reverse scores to healthy plant food groups and animal food groups. The score range of both hPDI and uPDI was 18 to 90 (highest adherence).

### Assessment of frailty

We used the FRAIL scale,^26^ which has previously been employed in the NHS.^10^ The FRAIL scale comprises five self‐reported frailty criteria: fatigue, low strength, reduced aerobic capacity, having several illnesses and a significant weight loss. In 1992, 1996, 2000, 2004, 2008 and 2012 the NHS participants completed the Medical Outcomes Study Short‐Form (SF‐36), a 36‐item‐questionnaire with eight health dimensions, including physical and mental components.^27^ From the SF‐36, we assessed the first three frailty criteria with the following questions: (1) for fatigue: ‘Did you have a lot of energy?,’ with replies ‘some of the time’ or ‘none of the time’ (in years 1992, 1996 and 2000), and the statement ‘I could not get going’ in an updated version of the SF‐36 (in 2004, 2008 and 2012), with responses ‘moderate amount’ or ‘all of the time’; (2) for low strength: ‘In a normal day, is your health a limitation to walk up 1 flight of stairs?,’ with responses ‘yes’ or ‘a lot’; and (3) for reduced aerobic capacity: ‘In a normal day, is your health a limitation to walk several blocks or several miles?,’ with responses ‘yes’ or ‘a lot.’ In addition, the illnesses criterion was assessed from the question, ‘In the last 2 years, have you had any of these physician‐diagnosed illnesses?’ We considered that this criterion was met when participants reported ≥5 of the following diseases: cancer, hypertension, type 2 diabetes, angina, myocardial infarction, stroke, congestive heart failure, asthma, chronic obstructive lung disease, arthritis, Parkinson's disease, kidney disease, and depression. Finally, the weight loss criterion was defined as a ≥5% decrease in weight reported over a 2‐year period. At the end of each follow‐up cycle, incident frailty was defined as having ≥3 criteria on the FRAIL scale. Missing data in three or more components was assumed as missing on frailty status and excluded. The FRAIL scale has been shown to be correlated (*r* = 0.62, *P* < 0.001) with the Fried scale,^28^ the most widely used scale for frailty assessment, which includes both self‐reported and performance‐based measures.

### Ascertainment of mortality

Deaths were identified from the state vital statistics records and the National Death Index or reported by families and the postal system. Follow‐up for mortality was more than 98% complete. For all deaths, we obtained copies of death certificates and, when appropriate, requested permission from the next of kin to review medical records to determine the causes of death, classified according to the International Classification of Diseases, Ninth Revision.

### Assessment of covariates

Biannually, we collected updated information on age, indicators of socio‐economic status (education level, census track income and husband's education), weight, smoking status, physical activity and medication use. Physical activity was assessed with a validated questionnaire.^29^ Body mass index (BMI), calculated as weight/height,^2^ was also assessed on each biennial questionnaire.

### Statistical analysis

Person‐years for each woman aged ≥60 years were calculated from the date of return of the questionnaire at baseline until the occurrence of frailty, death or the end of the study period (1 June 2014), whichever came first. Cumulative average of the indices was calculated over the follow‐up every 4 years since 1984 to better capture long‐term diet and reduce measurement error. Statistical tests were based on the a priori hypothesis that the quality of plant‐based diet is associated in opposite ways with the risk of frailty; thus, multiple testing was not necessary. However, we calculated the false discovery rate of 5% for two main comparisons using the Benjamini–Hoschberg procedure,^30^ and the associations remained the same. We evaluated the association between the hPDI and uPDI in quintiles and the risk of frailty using multivariable‐adjusted time‐varying Cox proportional hazards regression models, stratified by age in months and calendar time of each questionnaire cycle, to estimate hazard ratios (HRs) and 95% confidence intervals (CIs). Multivariable models included Model 1 adjusted for BMI (<25.0, 25.0–29.9, ≥30.0 kg/m^2^) at baseline (it was not updated because weight loss is part of the frailty outcome), socio‐economic status (that included education level, educational attainment of the participant's husband, marital status, employment status, census tract median income and census tract median home value) race, smoking status (never, past, and current 1–14, 15–24, and ≥25 cigarettes/day), alcohol intake (0, 1.0–4.9, 5.0–14.9, or ≥15.0 g/d), energy intake (quintiles of kcal/day), margarine intake (quintiles; because the fatty acid composition of margarine has changed over time from high‐trans fats to high‐unsaturated fats), and medication use (aspirin, postmenopausal hormone therapy, diuretics, β‐blockers, calcium channel blockers, ACE inhibitors, other blood pressure medication, statins and other cholesterol lowering drugs, insulin, and oral hypoglycaemic medication); and Model 2 further adjusted for physical activity because physical activity is related to the outcome. In secondary analyses we tested for linear trends by evaluating the quintile median values as a continuous variable and estimated the risk of frailty per 10‐unit increase in the hPDI and uPDI. We also assessed the association between plant‐based diets and each criterion of the FRAIL scale.

We conducted several sensitivity analyses to assess the robustness of results. First, we examined the association among those with none of the frailty criteria at baseline (namely robust individuals), or without cancer, cardiovascular disease (CVD), or diabetes. Second, based on our previous study where we found an inverse association between orange juices (which represented 65% of total juices) and risk of frailty, we calculated the scores without the juice item and adjusted for juice intake in the multivariable model. In addition, we created alternative healthy PDIs by assigning positive scores individually to fish and seafood and fermented dairy due to the evidence of the health benefits from the consumption of these food groups.^31^, ^32^ Also we assessed the individual contributions of healthy and less healthy plant foods and animal foods to the risk of frailty. And third, taking advantage of the repeated measures of diet over time, we evaluated the latency between the plant‐based diets and risk of frailty. We used diets scores reported at different latencies (i.e., 4–8 years, 8–12 years and 12–16 years) before the frailty occurrence. For example, in the 4–8 years analysis we used the diet scores in 1990 to evaluate the association with frailty risk in 1994–1998. This approach may help to account for the potential bias caused by changes in diet resulting from early signs of frailty. In addition, we used the most recent diet score assessment before the identification of frailty or simple update analysis (i.e., 0–4 year latency analysis) to assess the shorter term effect of PDI on frailty.

Analyses were performed using SAS software for UNIX, version 9.4 (SAS Institute Inc.).

## Results

Age‐adjusted baseline characteristics of participants according to quintiles of hPDI and uPDI are presented in *Table* *1*. Compared with participants with the lowest score in the hPDI, those with a higher score were leaner, more active, less likely to smoke, had higher intake of healthy plant foods and lower intake of less healthy plant foods, and had a smaller number of frailty criteria at baseline. Conversely, those with higher uPDI were also leaner but less active, more likely to smoke, and had higher intake of less healthy plant foods and lower of healthy plant foods and had a greater number of frailty criteria at baseline. Both indices showed lower energy intake across increasing quintiles.

**Table 1:** Age‐adjusted baseline characteristics according to quintiles of the plant‐based indices among women aged ≥60 years in the Nurses' Health Study^a^

	Healthful plant‐based diet index, quintile			Unhealthful plant‐based diet index, quintile		
	1	3	5	1	3	5
Participants, *n*	17 225	16 211	16 115	18 447	16 277	14 702
Dietary score	46.8 (3.1)	55.9 (1.3)	65.0 (2.9)	44.8 (3.1)	54.4 (1.1)	63.7 (3.1)
Age, year	63.9 (2.7)	64.2 (2.6)	64.4 (2.7)	64.1 (2.7)	64.2 (2.7)	64.2 (2.7)
BMI, kg/m^b^	26.2 (5.1)	25.6 (4.5)	25.0 (4.2)	26.2 (4.7)	25.5 (4.5)	25.3 (4.6)
Physical activity, MET‐h/week	15.9 (21)	18.3 (20.5)	23.1 (26.2)	23.5 (26.4)	19.4 (23.7)	14.6 (18.5)
Current smoker, %	13.4	10.5	7.2	8.1	11.0	13.7
Census tract median income (per 1000 USD)	58.6 (24.8)	58.9 (16.2)	59.2 (27.3)	62.0 (28.2)	59.0 (25.9)	55.4 (23.8)
Census tract median home value (per 10 000 USD)	16.3 (11.9)	16.9 (13.1)	18.3 (14.8)	19.1 (15.1)	17.0 (13.1)	14.9 (11.3)
Alcohol intake, g/day	5.8 (10.8)	5.5 (10.2)	4.6 (9.0)	5.7 (9.5)	5.5 (10.4)	4.4 (9.9)
Energy intake, kcal	2009 (464)	1695 (434)	1505 (392)	1989 (451)	1825 (441)	1495 (417)
Saturated fat (% of energy)	11.7 (2.5)	10.2 (2.3)	8.8 (2.2)	10.2 (2.4)	10.1 (2.5)	10.4 (2.7)
Monounsaturated fat (% of energy)	12.7 (2.3)	11.7 (2.5)	10.7 (2.9)	11.8 (2.5)	11.6 (2.7)	11.7 (2.7)
Polyunsaturated fat (% of energy)	5.8 (1.5)	5.9 (1.6)	5.9 (1.8)	6.2 (1.6)	5.9 (1.6)	5.5 (1.6)
Protein intake (% of energy)	17.7 (3.1)	18.9 (3.4)	19.5 (3.7)	20.2 (3.2)	18.9 (3.4)	17.2 (3.4)
Carbohydrate intake (% of energy)	49.3 (7.3)	51.2 (8.1)	53.9 (8.7)	49.9 (7.4)	51.3 (8.5)	53.0 (8.6)
Margarine intake, (s/day)	1.1 (1.0)	1.0 (0.9)	0.8 (0.8)	1.0 (0.9)	1.0 (0.9)	1.0 (0.9)
Vitamin B12, mcg	8.7 (5.8)	7.2 (5)	5.7 (4.3)	8.1 (5.0)	7.0 (5.1)	6.3 (5.3)
Calcium, mg	560.6 (333)	491.4 (322.1)	436.5 (314.4)	624.4 (338)	485 (320)	386.6 (285.7)
Healthy plant foods (s/day)	8.5 (3.2)	10.4 (3.7)	12.8 (4.1)	14.3 (3.9)	10.3 (2.9)	6.9 (2.5)
Whole grains	1.2 (1.1)	1.7 (1.3)	2.1 (1.5)	2.3 (1.5)	1.6 (1.3)	1.0 (1.0)
Fruits	1.5 (1.0)	1.8 (1.2)	2.3 (1.4)	2.5 (1.3)	1.9 (1.2)	1.3 (1.1)
Vegetables	2.6 (1.4)	3.1 (1.6)	3.7 (1.9)	4.3 (1.9)	3.1 (1.5)	2.0 (1.1)
Nuts	0.2 (0.3)	0.2 (0.3)	0.2 (0.4)	0.3 (0.4)	0.2 (0.3)	0.1 (0.2)
Legumes	0.4 (0.3)	0.4 (0.3)	0.5 (0.4)	0.6 (0.4)	0.4 (0.3)	0.3 (0.2)
Less healthy plant foods (s/day)	6.2 (2.6)	4.0 (2.0)	2.6 (1.6)	3.6 (2.1)	4.2 (2.4)	4.9 (2.6)
Refined grains	2.2 (1.6)	1.5 (1.2)	1.1 (1.0)	1.4 (1.2)	1.5 (1.3)	1.7 (1.5)
Potatoes	0.6 (0.4)	0.2 (0.4)	0.1 (0.2)	0.4 (0.3)	0.4 (0.3)	0.5 (0.4)
Sugar‐sweetened beverages	0.4 (0.6)	0.2 (0.4)	0.1 (0.2)	0.1 (0.3)	0.2 (0.4)	0.3 (0.6)
Sweets and desserts	1.9 (1.5)	1.2 (1.1)	0.8 (1.0)	1.1 (1.1)	1.3 (1.2)	1.5 (1.4)
Fruit juices	0.9 (0.8)	0.7 (0.7)	0.5 (0.7)	0.6 (0.7)	0.7 (0.7)	0.8 (0.8)
Animal foods (s/day)	5.9 (2.0)	4.5 (1.7)	3.5 (1.5)	6.0 (1.9)	4.5 (1.7)	3.4 (1.5)
Medication use, %^b^						
Aspirin	47.7	48.3	48.3	49.1	48.1	46.1
Postmenopausal hormone therapy	31.2	34.7	37.8	38.7	34.8	30.2
Diuretics	11.8	11.2	9.8	11.6	11.1	10.3
β‐blockers	15.2	13.8	11.9	12.9	14.2	14.8
Calcium channel blockers	10.1	10.6	9.7	12.3	12.1	12.6
ACE inhibitors	10.3	10.0	8.7	10.2	9.7	9.3
Other blood pressure medication	9.3	8.1	7.8	8.1	8.7	9.0
Statins	17.7	18.7	17.3	16.4	19.0	19.6
Other cholesterol medication	3.9	3.8	3.7	3.4	3.8	4.1
Insulin	1.5	1.7	2.2	2.3	1.6	1.2
Oral hypoglycaemic drugs	3.6	3.3	2.6	3.9	3.3	2.6
Number of frailty criteria, %						
0	75.0	78	81.8	80.1	77.7	75.8
1	19.5	18.0	14.6	16.1	18.4	18.9
2	5.5	4.0	3.5	3.9	3.9	5.4

Abbreviations: ACE, angiotensin‐converting enzyme; BMI, body mass index; MET, metabolic equivalent task.

^a^ *n* = values are means (*SD*s, standard deviation) unless otherwise indicated. Data, except age, were directly standardized to the age distribution of the entire cohort.

^b^ One or more times per week.

During 1 176 401 person‐years of follow‐up, we documented 12 910 cases of frailty. *Table* *2* shows the HRs (95% CI) for incident frailty according to quintiles of hPDI and uPDI. In multivariable‐adjusted analysis (Model 1), the hPDI was inversely associated with the risk of frailty: HR for the highest versus lowest quintile: 0.70 (0.66; 0.74), *P* trend <0.001. A 10‐unit increment in the hPDI was associated with a relative 19% lower risk of frailty. Conversely, a positive association was found between the uPDI and the risk of frailty: HR highest versus lowest quintile: 1.39 (1.31, 1.47), *P* trend <0.001. Further adjustment for physical activity at baseline attenuated the association, but it was still significant (Model 2): hPDI HR highest versus lowest quintile 0.77 (0.72, 0.81), *P* trend <0.001; uPDI HR highest versus lowest quintile: 1.24 (1.17, 1.32), *P* trend <0.001. Further adjustment for protein level did not change the result and no significant interaction was found for protein level (data no shown).

**Table 2:** Hazard ratios (95% confidence intervals) for frailty according to quintiles of plant‐based diet indices among women aged ≥60 y in the Nurses' Health Study

	Quintile 1	Quintile 2	Quintile 3	Quintile 4	Quintile 5	Per 10‐unit increase	*P* value for trend
Healthful plant‐based diet index (hPDI)							
Participants, *n*	18 447	17 131	16 277	15 677	14 702		
Person‐years	234 952	229 290	236 452	239 952	235 756		
Frailty cases	2779	2672	2687	2584	2188		
Age‐adjusted	Ref. 1.0	0.93 (0.89, 0.98)	0.87 (0.83, 0.92)	0.80 (0.75, 0.84)	0.65 (0.61, 0.68)	0.78 (0.75, 0.80)	<0.001
Multivariable Model 1	Ref. 1.0	0.94 (0.90, 1.00)	0.88 (0.83, 0.93)	0.81 (0.77, 0.86)	0.70 (0.66, 0.74)	0.81 (0.78, 0.83)	<0.001
Multivariable Model 2	Ref. 1.0	0.96 (0.91, 1.02)	0.92 (0.87, 0.97)	0.86 (0.81, 0.91)	0.77 (0.72, 0.81)	0.85 (0.82, 0.88)	<0.001
Unhealthful plant‐based diet index (uPDI)							
Participants, *n*	17 225	16 705	16 211	15 978	16 115		
Person‐years	239 041	230 904	238 420	231 686	236 350		
Frailty cases	2166	2352	2628	2667	3097		
Age‐adjusted	Ref. 1.0	1.07 (1.01, 1.14)	1.15 (1.09, 1.22)	1.15 (1.09, 1.22)	1.30 (1.23, 1.38)	1.14 (1.11, 1.17)	<0.001
Multivariable Model 1	Ref. 1.0	1.10 (1.03, 1.16)	1.20 (1.13, 1.27)	1.21 (1.14, 1.28)	1.39 (1.31, 1.47)	1.18 (1.15, 1.22)	<0.001
Multivariable Model 2	Ref. 1.0	1.06 (1.00, 1.12)	1.14 (1.08, 1.21)	1.13 (1.06, 1.20)	1.24 (1.17, 1.32)	1.12 (1.08, 1.15)	<0.001

*Note*: The multivariable Model 1 was adjusted for age (months), calendar time (4‐year intervals), body mass index (<25.0, 25.0–29.9, and ≥30.0 kg/m^2^) at baseline, socio‐economic status, smoking status (never, past and current; 1–14, 15–24, and ≥25 cigarettes per day), alcohol intake (0, 1.0–4.9, 5.0–14.9 or ≥15.0 g/day), energy intake (quintiles of kcal/day), margarine intake, and medication use (aspirin, postmenopausal hormone therapy, diuretics, β‐blockers, calcium channel blockers, ACE inhibitors, other blood pressure medication, statins and other cholesterol lowering drugs, insulin, and oral hypoglycaemic medication). The multivariable Model 2 was adjusted for Model 1 + physical activity.

We also evaluated the association between the PDI scores and each frailty criterion separately (*Table* *3*). Comparing the highest with the lowest quintile, the hPDI was inversely associated with each frailty criterion, being the strongest association for reduced aerobic capacity (HR 0.76 [0.72, 0.79]) followed by fatigue (HR 0.79 [0.77, 0.82]); and the weakest association was observed for having ≥5 illnesses (HR 0.90 [0.82, 0.99]). Contrarily, the uPDI was positively associated with each criterion except having ≥5 illnesses (*Table* *3*). The associations remained the same when using a false discovery rate of 5%.

**Table 3:** Hazard ratios (95% confidence intervals) for each frailty criterion according to quintiles of plant‐based diet indices among women aged ≥60 years in the Nurses' Health Study^a^

	Quintile 1	Quintile 2	Quintile 3	Quintile 4	Quintile 5	P Value for Trend
Healthful plant‐based diet index (hPDI)						
Fatigue, 34 493 cases						
Multivariable model	Ref 1.0	0.94 (0.91, 0.97)	0.92 (0.89, 0.95)	0.86 (0.83, 0.89)	0.79 (0.77, 0.82)	<0.001
Low strength, 12 263 cases						
Multivariable model	Ref 1.0	0.98 (0.92, 1.03)	0.90 (0.85, 0.95)	0.91 (0.86, 0.96)	0.85 (0.80, 0.90)	<0.001
Reduced aerobic capacity, 25 655 cases						
Multivariable model	Ref 1.0	0.94 (0.90, 0.97)	0.88 (0.84, 0.91)	0.83 (0.80, 0.86)	0.76 (0.73, 0.79)	<0.001
≥5 illnesses, 5051 cases						
Multivariable model	Ref 1.0	0.97 (0.89, 1.06)	0.97 (0.89, 1.06)	0.95 (0.87, 1.04)	0.91 (0.83, 1.01)	0.06
Weight loss ≥5 kg, 28 486 cases						
Multivariable model	Ref 1.0	0.93 (0.89, 0.96)	0.95 (0.92, 0.99)	0.93 (0.90, 0.97)	0.87 (0.84, 0.91)	<0.001
Unhealthful plant‐based diet index (uPDI)						
Fatigue, 34 493 cases						
Multivariable model	Ref 1.0	1.01 (0.98, 1.05)	1.08 (1.04, 1.12)	1.07 (1.04, 1.11)	1.16 (1.12, 1.20)	<0.001
Low strength, 12 263 cases						
Multivariable model	Ref 1.0	1.05 (0.99, 1.12)	1.14 (1.07, 1.21)	1.12 (1.04, 1.18)	1.21 (1.14, 1.29)	<0.001
Reduced aerobic capacity, 25 655 cases						
Multivariable model	Ref 1.0	1.09 (1.05, 1.14)	1.13 (1.08, 1.18)	1.17 (1.12, 1.22)	1.29 (1.24, 1.35)	<0.001
≥5 illnesses, 5051 cases						
Multivariable model	Ref 1.0	1.00 (0.91, 1.09)	1.05 (0.96, 1.15)	1.04 (0.95, 1.14)	0.99 (0.90, 1.09)	0.84
Weight loss ≥ 5 kg, 28 486 cases						
Multivariable model	Ref 1.0	1.00 (0.97, 1.05)	1.03 (0.99, 1.07)	1.06 (1.02, 1.10)	1.08 (1.03, 1.12)	<0.001

Abbreviations: hPDI, healthful plant‐based diet index, uPDI, unhealthful plant‐based diet index;

^a^ The multivariable‐adjusted model was adjusted for age (months), calendar time (4‐year intervals), body mass index (<25.0, 25.0–29.9 and ≥30.0 kg/m^2^) at baseline, socio‐economic status, smoking status (never, past and current; 1–14, 15–24, and ≥25 cigarettes per day), alcohol intake (0, 1.0–4.9, 5.0–14.9 or ≥15.0 g/day), energy intake (quintiles of kcal/day), margarine intake, and medication use (aspirin, postmenopausal hormone therapy, diuretics, β‐blockers, calcium channel blockers, ACE inhibitors, other blood pressure medication, statins and other cholesterol lowering drugs, insulin, and oral hypoglycaemic medication).

In sensitivity analyses, the associations remained similar among women without frailty criteria at baseline, and after excluding those with cancer, CVD and diabetes at baseline (*Table S2*). Likewise, the association remained unchanged with several modifications of the PDI (excluding fruit juices and scoring fish and dairy positively in the hPDI) (*Table S3*). Additionally, when we assessed the association between healthy plant foods, less healthy plant foods and animal foods intake in place of the PDI scores, the associations were significant and in the expected direction; the HR highest versus lowest quintile (95% CI) was_:_ 0.63 (0.59, 0.67) for healthy plant foods; 1.13 (1.05, 1.21) for less healthy plant foods; and 1.27 (1.17, 1.36) for animal foods (*Table S3*). We further assessed different latencies between the exposure and the outcome, and the associations tended to decrease with longer latency periods (*Table S5*).

Finally, the associations between 10‐unit increase in the PDI scores and risk of frailty persisted when we stratified by potential effect modifiers (age, smoking status, alcohol intake, physical activity, BMI, aspirin use and comorbidities (hypertension, hypercholesterolemia and diabetes) (*P* for interaction >0.05 in all cases) (*Table S6*).

## Discussion

In this large cohort of older women, a higher adherence to a healthy plant‐based diet was associated with lower risk of frailty, whereas an unhealthy plant‐based diet was associated with an increased risk. The association remained strong among those with no frailty criteria at baseline, after excluding those with prevalent diseases, using alternative versions of the PDI scores, using the consumption of healthy and unhealthy plant foods instead of the PDI scores, across different subgroups of participants, and in different latency analyses between the exposure and the outcome.

### Comparison with other studies

To our knowledge, no prior studies have specifically evaluated the association between the quality of plant‐based diets and risk of frailty. The EPIC‐Oxford cohort examined the association between non‐meat eaters (vegan and vegetarians) and risk of fractures and found that vegan and vegetarians had higher risk of fractures than meat eaters.^33^ However, the quality of the vegan and vegetarian diets was not evaluated, and this is essential because fractures are strongly associated with frailty and vice versa^34^ and also poor diet quality has been associated with risk of malnutrition, frailty and its adverse effects.^35^ Despite a lack of studies focusing on plant‐based diet quality and risk of frailty, previous evidence from large cohorts supports the role of diet quality in risk of frailty.^9^, ^10^, ^11^, ^12^, ^36^ In the NHS, we have previously reported that adherence to a Mediterranean diet, Dietary Approach to Stop Hypertension (DASH), and the alternate Healthy Eating Index‐2010 (AHEI‐2010) was associated with 13%, 7% and 10% lower risk of frailty, respectively.^11^ These diets include low consumption of animal foods; for example, higher consumption of red meat received lower scores. However, the plant‐based diets evaluated in our study differ from these other diets because the PDI negatively scores all animal foods and focus on the quality of the plant foods.

In this regard, despite the well‐recognized health effect of plant‐based diets during the life cycle,^1^, ^2^, ^3^, ^14^ concern has arisen due to the potential nutrition deficiencies involving vitamin B12, calcium or protein intake after an strict plant‐based diet. This is more important in older adults because a systematic review has shown that deficiencies in micronutrients (including vitamin D, carotenoids, vitamins B12, E or C) and macronutrients (including protein intake) are associated with increased risk of frailty.^35^ However, sources of most of those nutrients, with the exception of vitamin B12, are found in vegetable foods, which highlights the relevance of focusing on the quality of vegetarian diets. Indeed, our findings suggesting a protective effect of healthful plant foods versus less healthy foods intake or animal foods on the risk of frailty are in line with the results of another systematic review, where vegetarians or vegans diets had overall better quality than non‐vegetarians diets.^37^

Our results for each individual frailty criterion showed consistent inverse associations with higher adherence to hPDI and opposite associations for uPDI, which is in line with other studies analysing the quality of diet that share high intake of fruit, vegetables and whole grains.^11^ On the other hand, adequate protein intake has been associated with muscle mass, physical function, strength and frailty.^38^ In this regard, sources of dietary protein has been debated, with one position arguing that animal proteins increase muscle protein syntheses and digestibility compared with plant proteins.^39^ However, there is evidence that replacement of animal fats and protein sources, especially meat, with plant sources of protein is associated with lower risk of frailty^40^ and delay unhealthy ageing.^41^ Additionally, our recent findings showed that a higher intake of plant proteins but not animal or dairy protein, was associated with lower risk of frailty.^6^ This supports our findings that a dietary pattern emphasizing healthy and environmentally sustainable plant sources of protein can be achieved with no negative effect on muscle mass, stamina, weight and frailty. In fact, in our alternative PDI where we scored positively dairy, and fish did not alter the association, however, an intervention study with 40 Taiwanese individuals showed improvement in the frailty score when supplemented diet with skim milk powder and nuts.^42^ We only found a weaker association with the criterion ≥5 illnesses, however, our sensitivity analysis excluding those with cancer, CVD and diabetes or among robust participants at baseline remained strong.

### Potential underlying mechanisms

The mechanisms underlying our findings may involve specific components of the hPDI, such as antioxidants, unsaturated fatty acids, dietary fibre and micronutrients including vitamins and carotenoids.^1^, ^14^ These components have been linked to lower indicators of inflammation, and they may help to counteract the state of low‐grade chronic inflammation that underlies frailty. In addition, higher consumption of fruits and vegetables that have high antioxidant capacity may reduce risk of frailty.^21^ Furthermore, these and other components of a healthful plant‐based diet, such as plant oils or whole grains, as well as the overall hPDI have been linked to lower incidence of CVD, obesity or diabetes,^15^, ^17^, ^18^ conditions that are risk factors for frailty. In contrast, refined carbohydrates, sugary‐sweetened beverages and processed meats with high pro‐inflammatory properties have been associated with frailty.^22^ Finally, an optimal protein intake is related to higher muscle mass and bone mineralization, which may postpone the onset of frailty in older adults.^4^

Substantial evidence supports the beneficial effects of physical activity on physical function and frailty in older adults.^43^ Our analysis adjusting for physical activity only slightly attenuated the protective association between hPDI and frailty, suggesting that this association is not explained by the greater physical activity of those in the highest quintiles of the hPDI.

### Strengths and limitations

Strengths of our study include the large sample size, the follow‐up over more than 22 years, and the use of repeated and updated measures of diet and the covariates all help to minimize bias. Limitations of our study include that diet was self‐reported, which may lead to measurement error and misclassification. However, the use of a FFQ that has been validated against biomarkers and diet records,^24^ and the repeated measurement of habitual diet over time to obtain a cumulative average of the PDI scores, can reduce this error. Also, because we focused on overall long‐term plant‐based diet quality, we did not evaluate the relation of specific plant‐for‐animal substitutions to the risk of frailty; however, our recent analysis suggested that replacing processed red meats with nuts and legumes was associated with 26% and 13% lower risk of frailty.^44^ In addition, all animal foods were scored negatively whereas evidence suggests that the consumption of dairy as a main source of calcium intake and other foods such as fish may have a null effect on fractures, or CVD linked to frailty; however, our sensitivity analyses scoring those items positively did not alter the findings. Despite adjusting for multiple confounders including socio‐economic, lifestyles, medication and chronic diseases, residual confounding cannot be rule out. In addition, reverse causation cannot be excluded although the results from the latency analysis are consistent with the main results but attenuated. Finally, our study comprises only female nurses; thus, generalization to other populations should be done cautiously.

## Conclusion

A healthful plant‐based diet rich in fruits, vegetables, whole grains, nuts and legumes was associated with lower risk of frailty, whereas an unhealthful plant‐based diet characterized by higher amounts of juices, refined grains or sugar‐sweetened beverages was linked to higher risk. These findings support a global shift towards healthful plant‐based diets in older adults for preventing frailty and highlight the importance of the quality of the plant‐foods to achieve this goal.

## Conflict of interest

Mercedes Sotos‐Prieto, Ellen A. Struijk, Teresa T. Fung, Fernando Rodriguez‐Artalejo, Walter C. Willett, Frank B. Hu and Esther Lopez‐Garcia declare that they have no conflict of interest.

## Supporting information


**Table S1.** Food items and criteria for scoring each plant‐based diet indices (from the 1990 NHS food frequency questionnaire).
**Table S2.** Hazard Ratios (95% CIs) for frailty according to quintiles of Plant‐based diets among women who 1) had no frailty criteria at baseline and 2) had no cancer, cardiovascular disease or diabetes at baseline.
**Table S3.** Hazard Ratios (95% CIs) for frailty according to quintiles of modified versions of Plant‐Based Diet Indices among women aged ≥60 y in the Nurses' Health Study*
**Table S4.** Hazard Ratios (95% CIs) for frailty according to quintiles of food categories among women aged ≥60 y in the Nurses' Health Study
**Table S5.** Hazard Ratios (95% CIs) for frailty according to quintiles of Plant‐Based Diet Indices with different latency periods using data among women aged ≥60 y in the Nurses' Health Study*
**Table S6.** Hazard Ratios (95% CIs) for frailty according to quintiles of Plant‐Based Diet Indices across subgroups of women aged ≥60 y in the Nurses' Health Study*
**Figure S1.** Participant flow chart
