# Supplementary material for: Association between the quality of plant‐based diets and risk of frailty
Source: J Cachexia Sarcopenia Muscle. 2022 Sep 30;13(6):2854–62. doi: 10.1002/jcsm.13077 (PMC9745455; doi:10.1002/jcsm.13077)

Supplementary table 1. Food items and criteria for scoring each plant-based diet indices (from the 1990 NHS food frequency questionnaire).

		hPDI	uPDI
Healthy plant foods			
Whole grains	Whole grain breakfast cereal, other cooked breakfast cereal, cooked oatmeal, dark bread, brown rice, other grains, bran, wheat germ, popcorn	Positive scores	Reverse scores
Fruits	Raisins or grapes, prunes, bananas, cantaloupe, watermelon, avocado, fresh apples, applesauce, fresh pears, canned pears, oranges, grapefruit, strawberries, blueberries, peaches or apricots or plums, canned peaches or apricots, other canned fruit	Positive scores	Reverse scores
Vegetables	Tomatoes, tomato juice, tomato sauce, red chili sauce, broccoli, cauliflower, cooked cabbage, coleslaw or uncooked cabbage, Brussels sprouts, raw carrots, cooked carrots, mixed vegetables, yams or sweet potatoes, yellow or winter squash, eggplant or zucchini or other summer squash, kale or mustard or chard greens, cooked spinach, raw spinach, iceberg or head lettuce, romaine or leaf lettuce, green pepper, cucumber, celery, mushrooms, alfalfa sprouts, corn	Positive scores	Reverse scores
Nuts	Nuts, peanut butter	Positive scores	Reverse scores
Legumes	String beans, tofu or soybeans, beans or lentils, peas or lima beans	Positive scores	Reverse scores
Vegetable oil	Vegetable oil, oil-based salad dressing	Positive scores	Reverse scores
Tea and coffee	Tea, coffee, decaffeinated coffee or non-caffeinated tea	Positive scores	Reverse scores
Less healthy plant foods			

Refined grains	Refined grain breakfast cereal, white bread, English muffins or bagels or rolls, muffins or biscuits, white rice, pancakes or waffles, crackers, pasta	Reverse scores	Positive scores
Potatoes	French fries, baked or boiled or mashed potatoes, potato or corn chips	Reverse scores	Positive scores
Sugar-sweetened beverages	Soda with sugar, noncarbonated fruit drinks with sugar	Reverse scores	Positive scores
Fruit juices	Apple cider (nonalcoholic) or juice, orange juice, grapefruit juice, other fruit juice	Reverse scores	Positive scores
Sweets and desserts	Chocolates, candy bars, candy without chocolate, cookies (home-baked and ready-made), brownies, doughnuts, cake (home-made and ready-made), sweet roll or coffee cake or other pastry, pie (home-baked and ready-made), jams or jellies or preserves or syrup or honey	Reverse scores	Positive scores
Animal foods			
Animal fat	Butter added to food, butter or lard used for cooking	Reverse scores	Reverse scores
Dairy	Skim low fat milk, whole milk, cream, sour cream, sherbet, ice cream, yogurt, cottage or ricotta cheese, cream cheese, other cheese	Reverse scores	Reverse scores
Eggs	Eggs	Reverse scores	Reverse scores
Fish and seafood	Canned tuna, dark meat fish, other fish, shrimp or lobster or scallops	Reverse scores	Reverse scores
Meat	Chicken or turkey with skin, chicken or turkey without skin, bacon, hot dogs, processed meats, hamburger, liver, beef or pork or lamb as a mixed dish, beef or pork or lamb as a main dish	Reverse scores	Reverse scores
Miscellaneous animal-based foods	Pizza, chowder or cream soup, mayonnaise or other creamy salad dressing	Reverse scores	Reverse scores

Abbreviations: hPDI, healthful plant-based diet index, uPDI, unhealthful plant-based diet index; NHS, Nurses' Health Study.

Supplementary Table 2. Hazard Ratios (95% CIs) for frailty according to quintiles of Plant-based diets among women who 1) had no frailty criteria at baseline and 2) had no cancer, cardiovascular disease or diabetes at baseline.

	Quintile 1	Quintile 2	Quintile 3	Quintile 4	Quintile 5	*P* Value for Trend
**No frailty criteria at baseline**						
**Healthful Plant-Based Diet Index**						
Cases/persons-years	2779/234952	2672/229290	2687/236452	2584/239952	2188/235756	
Multivariable-adjusted model	Ref. 1.0	0.94 (0.90, 1.00)	0.88 (0.83, 0.93)	0.81 (0.77, 0.86)	0.70 (0.66, 0.74)	<0.001
**Unhealthful Plant-Based Diet Index**						
Cases/persons-years	2166/239041	2352/230904	2628/238420	2667/231686	2097/236350	
Multivariable-adjusted model	Ref. 1.0	1.09 (1.03, 1.16)	1.20 (1.13, 1.27)	1.21 (1.14, 1.28)	1.39 (1.30, 1.47)	<0.001
**Excluding those with cancer, CVD or diabetes at baseline**						
**Healthful Plant-Based Diet Index**						
Cases/persons-years	2277/206908	2241/207412	2189/209667	2126/212293	1781/208750	
Multivariable-adjusted model	Ref. 1.0	0.93 (0.87, 0.98)	0.87 (0.82, 0.92)	0.81 (0.76, 0.86)	0.68 (0.64, 0.73)	<0.001
**Unhealthful Plant-Based Diet Index**						
Cases/persons-years	1758/212275	1913/204463	2164/212723	2187/204283	2592/211285	
Multivariable-adjusted model	Ref. 1.0	1.08 (1.01, 1.15)	1.18 (1.11, 1.26)	1.22 (1.14, 1.30)	1.39 (1.30, 1.48)	<0.001

The multivariable-adjusted model was adjusted for age (months), calendar time (4-y intervals), body mass index (<25.0, 25.0-29.9, ≥30.0 kg/m^2) at baseline, socioeconomic status, smoking status (never, past, and current 1-14, 15-24, and ≥25 cigarettes/day), alcohol intake (0, 1.0-4.9, 5.0-14.9, or ≥15.0 g/d), energy intake (quintiles of kcal/d), margarine intake, and medication use (aspirin, postmenopausal hormone

therapy, diuretics, β-blockers, calcium channel blockers, ACE inhibitors, other blood pressure medication, statins and other cholesterol lowering drugs, insulin, oral hypoglycemic medication).

Supplementary Table 3. Hazard Ratios (95% CIs) for frailty according to quintiles of modified versions of Plant-Based Diet Indices among women aged ≥60 y in the Nurses' Health Study*

	Quintile 1	Quintile 2	Quintile 3	Quintile 4	Quintile 5	*P* Value for Trend
**Healthful Plant-Based Diet Index without fruit juices**						
Cases/persons-years	2776/235739	2690/232980	2703/235086	2569/237002	2172/235595	
Multivariable-adjusted model	Ref. 1.0	0.92 (0.87, 0.97)	0.88 (0.83, 0.93)	0.80 (0.75, 0.84)	0.68 (0.64, 0.72)	<0.001
**Healthful Plant-Based Diet Index without orange juices**						
Cases/persons-years	2717/235470	2672/231823	2718/239519	2584/233340	2219/236249	
Multivariable-adjusted model	Ref. 1.0	0.93 (0.88, 0.98)	0.88 (0.84, 0.93)	0.82 (0.78, 0.87)	0.70 (0.65, 0.74)	<0.001
**Unhealthful Plant-Based Diet Index without fruit juices**						
Cases/persons-years	2164/236143	2424/235312	2564/238351	2724/231209	3034/235386	
Multivariable-adjusted model	Ref. 1.0	1.12 (1.06, 1.19)	1.22 (1.14, 1.29)	1.29 (1.22, 1.37)	1.45 (1.36, 1.55)	<0.001
**Unhealthful Plant-Based Diet Index without orange juices**						
Cases/persons-years	2153/234649	2449/236409	2579/237630	2731/233834	2998/233879	
Multivariable-adjusted model	Ref. 1.0	1.11 (1.05, 1.18)	1.19 (1.12, 1.26)	1.24 (1.17, 1.32)	1.38 (1.30, 1.47)	<0.001
**Healthful Plant-Based Diet Index with fish (positive score)**						
Cases/persons-years	2827/235582	2862/235260	2654/233856	2492/234953	2075/236749	

Multivariable-adjusted model	Ref.	0.95 (0.90, 1.00)	0.88 (0.83, 0.93)	0.79 (0.75, 0.83)	0.68 (0.64, 0.72)	<0.001
**Healthful Plant-Based Diet Index with yogurt (positive score)**						
Cases/persons-years	2797/233344	2800/234784	2676/23668	2503/235115	2134/236490	
Multivariable-adjusted model	Ref.	0.95 (0.90, 1.00)	0.88 (0.83, 0.93)	0.80 (0.76, 0.85)	0.70 (0.66, 0.74)	<0.001

The multivariable-adjusted model was adjusted for age (months), calendar time (4-y intervals), body mass index (<25.0, 25.0-29.9, ≥30.0 kg/m^2) at baseline, socioeconomic status, smoking status (never, past, and current 1-14, 15-24, and ≥25 cigarettes/day), alcohol intake (0, 1.0-4.9, 5.0-14.9, or ≥15.0 g/d), energy intake (quintiles of kcal/d), margarine intake, and medication use (aspirin, postmenopausal hormone therapy, diuretics, β-blockers, calcium channel blockers, ACE inhibitors, other blood pressure medication, statins and other cholesterol lowering drugs, insulin, oral hypoglycemic medication).

Supplementary Table 4. Hazard Ratios (95% CIs) for frailty according to quintiles of food categories among women aged ≥60 y in the Nurses' Health Study

	Quintile 1	Quintile 2	Quintile 3	Quintile 4	Quintile 5	*P* Value for Trend
**Healthful plant-food intake**						
Cases/persons-years	3088/233758	2774/234816	2561/235446	2353/235978	2134/236376	
Multivariable-adjusted model	Ref. 1.0	0.87 (0.83, 0.92)	0.78 (0.73, 0.82)	0.71 (0.67, 0.75)	0.64 (0.60, 0.68)	<0.001
**Less healthy plant food intake**						
Median (range)	2.1 (0, 2.7)	3.1 (2.4, 3.6)	3.9 (3.3, 4.5)	4.9 (4.2, 5.7)	6.7 (5.3, 21.4)	
Cases/persons-years	2617/235235	2425/235496	2525/235496	2627/235298	2716/234599	
Multivariable-adjusted model	Ref. 1.0	0.93 (0.88, 0.99)	0.99 (0.93, 1.05)	1.07 (1.00, 1.14)	1.12 (1.04, 1.20)	<0.001
**Animal food intake**						
Median (range)	2.8 (0, 3.4)	3.8 (3.4, 4.3)	4.6 (4.2, 5.1)	5.4 (4.9, 6.2)	6.8 (5.9, 20.2)	
Cases/persons-years	2490/235500	2544/235519	2605/235263	2603/235544	2668/234576	
Multivariable-adjusted model	Ref. 1.0	1.08 (1.02, 1.14)	1.13 (1.06, 1.21)	1.19 (1.11, 1.27)	1.27 (1.17, 1.37)	<0.001

The multivariable-adjusted model was adjusted for age (months), calendar time (4-y intervals), body mass index (<25.0, 25.0-29.9, ≥30.0 kg/m^2) at baseline, socioeconomic status, smoking status (never, past, and current 1-14, 15-24, and ≥25 cigarettes/day), alcohol intake (0, 1.0-4.9, 5.0-14.9, or ≥15.0 g/d), energy intake (quintiles of kcal/d), margarine intake, and medication use (aspirin, postmenopausal hormone therapy, diuretics, β-blockers, calcium channel blockers, ACE inhibitors, other blood pressure medication, statins and other cholesterol lowering drugs, insulin, oral hypoglycemic medication).

Supplementary Table 5. Hazard Ratios (95% CIs) for frailty according to quintiles of Plant-Based Diet Indices with different latency periods using data among women aged ≥60 y in the Nurses' Health Study*

	0-4 y latency	4-8 y latency	8-12 y latency	12-16 y latency
**Healthful plant-based diet index**				
Quintile 1	Ref 1.0	Ref 1.0	Ref 1.0	Ref 1.0
Quintile 2	0.84 (0.79, 0.89)	0.94 (0.89, 0.99)	0.93 (0.88, 0.98)	0.98 (0.92, 1.05)
Quintile 3	0.81 (0.77, 0.86)	0.88 (0.83, 0.93)	0.88 (0.83, 0.93)	0.91 (0.86, 0.97)
Quintile 4	0.74 (0.70, 0.78)	0.80 (0.76, 0.85)	0.81 (0.77, 0.86)	0.86 (0.80, 0.91)
Quintile 5	0.62 (0.59, 0.66)	0.70 (0.66, 0.74)	0.74 (0.70, 0.79)	0.75 (0.70, 0.81)
p trend	<0.001	<0.001	<0.001	<0.001
**Unhealthful plant-based diet index**				
Quintile 1	Ref 1.0	Ref 1.0	Ref 1.0	Ref 1.0
Quintile 2	1.10 (1.03, 1.18)	1.10 (1.03, 1.16)	1.07 (1.00, 1.13)	1.02 (0.95, 1.09)
Quintile 3	1.20 (1.12, 1.27)	1.19 (1.13, 1.26)	1.09 (1.03, 1.16)	1.08 (1.01, 1.15)
Quintile 4	1.31 (1.23, 1.40)	1.19 (1.13, 1.27)	1.13 (1.06, 1.20)	1.18 (1.09, 1.26)
Quintile 5	1.55 (1.45, 1.65)	1.33 (1.25, 1.41)	1.23 (1.16, 1.31)	1.22 (1.14, 1.30)
p trend	<0.001	<0.001	<0.001	<0.001

*The multivariable-adjusted model was adjusted for age (months), calendar time (4-y intervals), body mass index (<25.0, 25.0-29.9, ≥30.0 kg/m2) at baseline, socioeconomic status, smoking status (never, past, and current 1-14, 15-24, and ≥25 cigarettes/day), alcohol intake (0, 1.0-4.9, 5.0-14.9, or ≥15.0 g/d), energy intake (quintiles of kcal/d), margarine intake, and medication use (aspirin, postmenopausal hormone therapy, diuretics, β-blockers, calcium channel blockers, ACE inhibitors, other blood pressure medication, statins and other cholesterol lowering drugs, insulin, oral hypoglycemic medication).

Supplementary Table 6. Hazard Ratios (95% CIs) for frailty according to quintiles of Plant-Based Diet Indices across subgroups of women aged ≥60 y in the Nurses' Health Study*

	**Per 10-unit increase Healthful plant-based diet index**	**Per 10-unit increase Unhealthful plant-based diet index**
Age, years		
<65	0.83 (0.75, 0.92)	1.19 (1.08, 1.31)
≥65	0.80 (0.77, 0.82)	1.19 (1.16, 1.23)
Smoking status		
Never	0.83 (0.80, 0.85)	1.18 (1.14, 1.22)
Ever	0.86 (0.76, 0.97)	1.15 (1.02, 1.29)
Alcohol intake		
Non-drinker	0.79 (0.75, 0.83)	1.23 (1.17, 1.29)
Drinker	0.82 (0.79, 0.86)	1.15 (1.11, 1.20)
Protein intake		
<1.2 g/kg/day	0.81 (0.74, 0.89)	1.19 (1.09, 1.31)
≥1.2 g/kg/day	0.81 (0.78, 0.83)	1.19 (1.15, 1.22)
Physical Activity		
≤150 min	0.86 (0.82, 0.89)	1.13 (1.09, 1.18)
>150 min	0.81 (0.77, 0.84)	1.17 (1.11, 1.23)
Body mass index (Kg/m^2)		
<25	0.77 (0.73, 0.81)	1.29 (1.22, 1.36)
≥25	0.80 (0.77, 0.83)	1.12 (1.08, 1.16)
Aspirin use		
No	0.81 (0.79, 0.84)	1.18 (1.14, 1.21)
Yes	0.65 (0.55, 0.77)	1.36 (1.16, 1.58)
Hypertension		
No	0.78 (0.75, 0.81)	1.25 (1.20, 1.30)
Yes	0.85 (0.81, 0.89)	1.11 (1.06, 1.16)
Hypercholesterolemia		

No	0.79 (0.75, 0.82)	1.21 (1.16, 1.26)
Yes	0.83 (0.79, 0.87)	1.15 (1.10, 1.20)
Diabetes		
No	0.80 (0.77, 0.82)	1.20 (1.16, 1.24)
Yes	0.88 (0.80, 0.96)	1.11 (1.02, 1.21)

*The multivariable-adjusted model was adjusted for age (months), calendar time (4-y intervals), body mass index (<25.0, 25.0-29.9, ≥30.0 kg/m^2) at baseline, socioeconomic status, smoking status (never, past, and current 1-14, 15-24, and ≥25 cigarettes/day), alcohol intake (0, 1.0-4.9, 5.0-14.9, or ≥15.0 g/d), energy intake (quintiles of kcal/d), margarine intake, and medication use (aspirin, postmenopausal hormone therapy, diuretics, β-blockers, calcium channel blockers, ACE inhibitors, other blood pressure medication, statins and other cholesterol lowering drugs, insulin, oral hypoglycemic medication).

eFigure 1 Participant flow chart

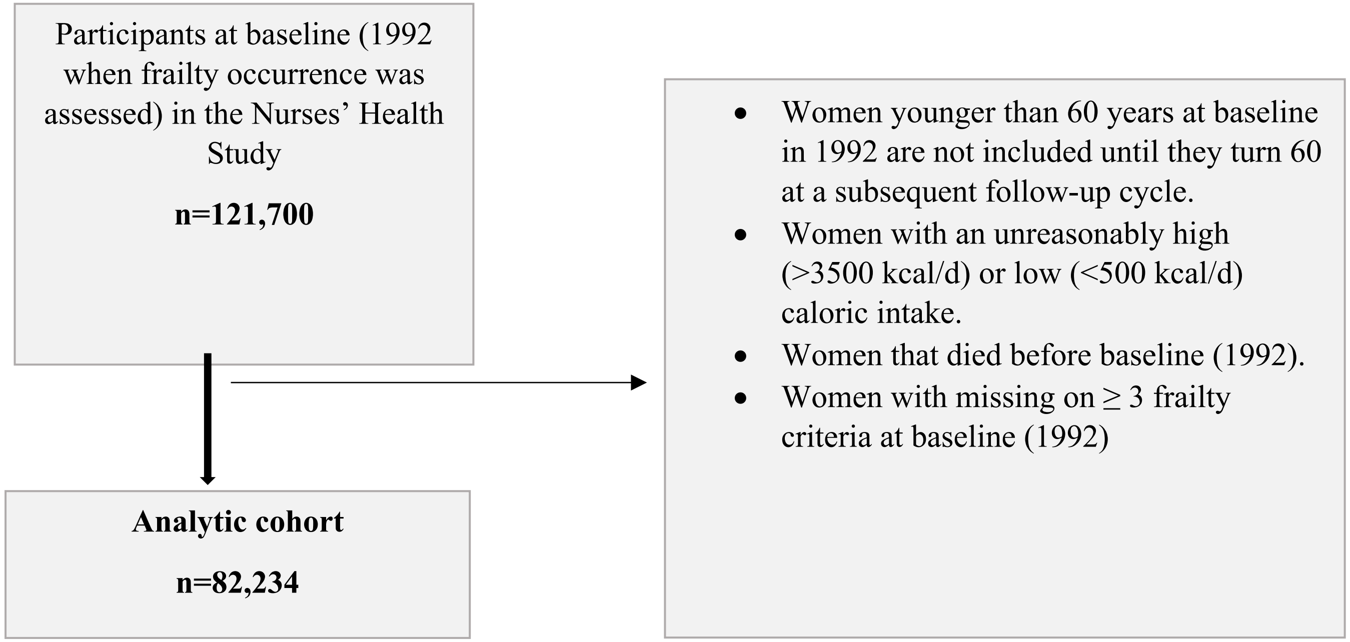

Supplement: Supplementary file 1 — Table S1. Food items and criteria for scoring each plant‐based diet indices (from the 1990 NHS food frequency questionnaire). Table S2. Hazard Ratios (95% CIs) for frailty according to quintiles of Plant‐based diets among women who 1) had no frailty criteria at baseline and 2) had no cancer, cardiovascular disease or diabetes at baseline. Table S3. Hazard Ratios (95% CIs) for frailty according to quintiles of modified versions of Plant‐Based Diet Indices among women aged ≥60 y in the Nurses' Health Study* Table S4. Hazard Ratios (95% CIs) for frailty according to quintiles of food categories among women aged ≥60 y in the Nurses' Health Study Table S5. Hazard Ratios (95% CIs) for frailty according to quintiles of Plant‐Based Diet Indices with different latency periods using data among women aged ≥60 y in the Nurses' Health Study* Table S6. Hazard Ratios (95% CIs) for frailty according to quintiles of Plant‐Based Diet Indices across subgroups of women aged ≥60 y in the Nurses' Health Study* Figure S1. Participant flow chart [file JCSM-13-2854-s001.pdf]
